# Review: chitosan-based biopolymers for anion-exchange membrane fuel cell application

**DOI:** 10.1098/rsos.230843

**Published:** 2023-11-08

**Authors:** Bauyrzhan Myrzakhmetov, Aktilek Akhmetova, Aiman Bissenbay, Mirat Karibayev, Xuemiao Pan, Yanwei Wang, Zhumabay Bakenov, Almagul Mentbayeva

**Affiliations:** ^1^ Center for Energy and Advanced Materials Science, National Laboratory Astana, Nazarbayev University, 53 Kabanbay Batyr Avenue, Astana, Kazakhstan; ^2^ Department of Chemical and Materials Engineering, School of Engineering and Digital Sciences, Nazarbayev University, 53 Kabanbay Batyr Avenue, Astana, Kazakhstan

**Keywords:** anion exchange membrane fuel cells, chitosan, composite membranes

## Abstract

Chitosan (CS)-based anion exchange membranes (AEMs) have gained significant attention in fuel cell applications owing to their numerous benefits, such as environmental friendliness, flexibility for structural alteration, and improved mechanical, thermal and chemical durability. This study aims to enhance the cell performance of CS-based AEMs by addressing key factors including mechanical stability, ionic conductivity, water absorption and expansion rate. While previous reviews have predominantly focused on CS as a proton-conducting membrane, the present mini-review highlights the advancements of CS-based AEMs. Furthermore, the study investigates the stability of cationic head groups grafted to CS through simulations. Understanding the chemical properties of CS, including the behaviour of grafted head groups, provides valuable insights into the membrane’s overall stability and performance. Additionally, the study mentions the potential of modern cellulose membranes for alkaline environments as promising biopolymers. While the primary focus is on CS-based AEMs, the inclusion of cellulose membranes underscores the broader exploration of biopolymer materials for fuel cell applications.

## 1. Introduction

**Figure 1.**
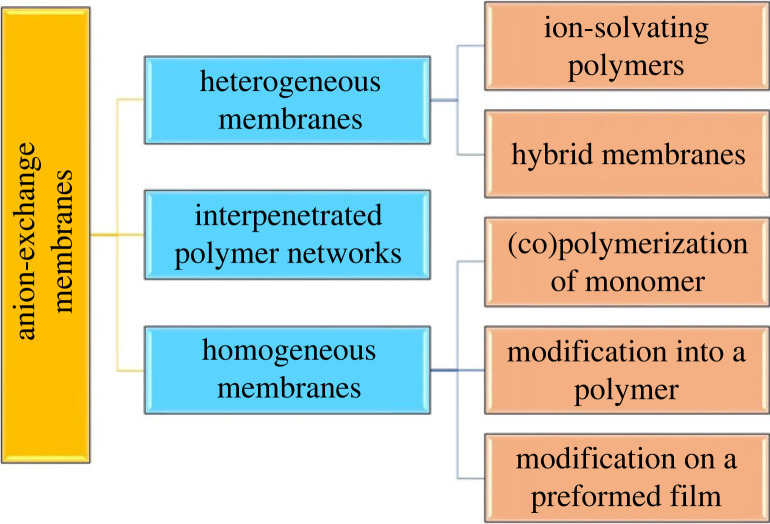
Schematic of various polymer electrolyte membranes.

The exploration and use of environmentally safe power sources as alternatives to fossil fuels are critical steps in addressing the depletion of petroleum reserves, preventing global warming and reducing pollution. Transitioning to sustainable energy is not only environmentally beneficial but also offers numerous economic and societal advantages [1–3]. In this regard, fuel cells, which possess efficient energy conversion, renewable resources and increased awareness regarding the toxic by-products amid operation, have gained significant interest in the electrical power market [4–6]. Various types of fuel cells are designed depending on the nature of fuels used and ion-conductive media. The direct hydrogen and methanol fuel cells are particularly interesting owing to their minimal environmental impact, and liquid methanol is more appealing in terms of organization, affordability and safety, particularly for transportation purposes [7]. In compliance with demanding thermal, mechanical and chemical requirements, special attention was given to developing the low-temperature proton and anion exchange membrane fuel cells (PEMFC and AEMFC, respectively). AEMFCs differ from PEMFCs in terms of operating conditions and used electrodes. The now-common proton exchange membranes (PEMs) perform in acidic conditions, and anion exchange membranes (AEMs) operate in an alkaline medium. Therefore, owing to the different ion transfer mechanisms, transition metal (TM) catalysts can be used in AEMFCs, revealing even faster oxygen reduction kinetics compared to PEMFC’s noble metal catalysts [8,9]. From a commercial perspective, this substitution significantly reduces the cost of fuel cells. Furthermore, AEMFC can be operated using other fuels, such as urea and biogas, which decompose into more ecologically friendly products than hydrocarbons. The researchers are suggesting that although PEMFCs have some drawbacks, their overall performance is still better than that of alkaline fuel cells (AFCs). However, they also suggest that there is room for improvement in the development and application of AEMs which are a type of membrane used in AFCs. Improving the electrical and physico-chemical properties of AEMs, such as their ionic conductivity and stability, could help to overcome some of the drawbacks of AFCs and make them more competitive with PEMFCs [10–13]. The AEMs could be classified according to their structures or preparation procedures. There are many articles on AEMs preparation, that describe the synthesis via different routes: (i) alkali-doped polymer blends, (ii) hybrid membranes by sol-gel method, (iii) polymers with (semi)interpenetrated systems, (iv) (co)polymerization, and (v) chemical and radio-grafting modification [7]. Homogeneous polymer membranes are typically prepared via blending methods, where the polymer matrix is doped with an alkali metal hydroxide. These membranes are typically easy to prepare and exhibit good chemical stability, making them a popular choice for use in fuel cells and other electrochemical devices. On the other hand, heterogeneous membranes are prepared via the interpenetration of two different polymer matrices, which can provide greater control over the membrane structure and properties. These membranes can be further classified based on their composition, with semi-interpenetrating polymer networks (semi-IPNs) containing only partially interpenetrating polymer chains, while interpenetrating polymer networks (IPNs) contain fully interpenetrating polymer chains, as shown in figure 1. Depending on the type and purpose of modification, it could either be altered before or after forming the membrane. For example, in polymer blends, one of the components might be grafted with cationic head groups before the film is prepared. By contrast, the cross-linking process could be performed after the film formation, depending on the polymer types. The heterogeneous membranes could be divided further into ion-solvating and composite ones.

The transportation of OH^−^ anions formed at the cathode in AEMFCs is promoted by polymers, which consist of a hydrophobic side chain with cations and water. The polymer backbone is an essential component of the AEM that affects its properties. It provides strength and stability, and positively charged ions can enhance hydroxide conductivity, which involves the functionalization of membranes, e.g. grafting ion-conducting groups on polymer backbone chains [14]. Chitosan (CS) contains amino groups (-NH_2_) that can be protonated or deprotonated depending on the pH of the surrounding environment. In alkaline conditions, the amino groups can attract and exchange hydroxide ions (OH^−^) with other ions in the electrolyte solution. This ion exchange mechanism allows for the transport of OH^−^ ions through the membrane. The conduction of OH^−^ in membranes is also facilitated by conductive cation aggregation zones. These zones are regions within the membrane where cations can aggregate and enable the movement of OH^−^ ions. Maintaining the continuity of these zones is crucial for efficient ion conduction [15–17]. Additionally, the incorporation of specific hydroxide-conductive fillers or additives can enhance OH^−^ conduction in CS-based membranes. For example, the addition of inorganic hydroxide-conductive materials, such as hydrated metal oxides or hydroxides, can promote OH^−^ transport through the membrane. These fillers can provide pathways for OH^−^ ions to migrate and increase the overall ionic conductivity of the CS membrane [18].

The polymer backbone should satisfy the following parameters to be a component of AEM in AFC [12]: chemical and mechanical stability in alkaline media; thermal stability at elevated operating temperatures; hydroxide conductivity (around 0.1 S cm^−1^); and capacity to insulate electrons; cost-effectiveness.

Over the last few decades, several approaches have been described for selecting suitable polymers for synthesizing AEMs to improve their thermal, chemical and electrochemical properties. Currently, most AEMs comprise synthetic polymers, such as poly(phenylene oxide) (PPO) [19,20], polysulfone (PSF) [21,22], poly(fluorene) [23], poly(vinyl alcohol) (PVA) [24–27], poly(vinylidene fluoride) (PVDF) [28] based ones and enhanced performance in fuel cells. For example, PPO membrane modified with quaternary ammonium groups (QA) exhibited ionic conductivity (IC) of above 35 mS cm^−1^ (75°C) and ion-exchange capacities (IEC) of around 2−3 mmol g^−1^ [19,20]. Additionally, polymeric blends were used to enhance membrane properties and functionality, such as the hydroxide transport ability of a cross-linked imidazolium (Im)-PVA and brominated PPO blend, which achieved an ionic conductivity of 78.8 mS cm^−1^ and an ion-exchange capacity of 1.54 mmol g^−1^ at 80°C [25].

PVA is a synthetic polymer with a structure consisting of a carbon backbone and hydroxyl functional groups [29]. PVA’s hydrophilicity, film-forming ability, the presence of reactive groups, and compatibility with other polymers and inorganic compounds make it a versatile choice for designing AEMs with a range of desirable properties for electrochemical applications, particularly in fuel cells and related technologies [30]. Futhermore, PVA can significantly contribute to the success of fibre spinning processes by reducing repulsive forces within charged polymer solutions, improving solution stability, and leading to the production of high-quality fibre membranes [31].

On the other hand, CS is a biopolymer derived from chitin, which is a natural polymer found in the shells of crustaceans like shrimp and crabs, as well as in the cell walls of fungi. It is an interesting and versatile substance with a range of potential applications owing to its unique properties (table 1).

**Table 1.** The major differences between PVA and CS polymer matrices.

polymers	main characteristics	ref.
polyvinyl alcohol (PVA)	easy fabrication, highly water-soluble, high tensile strength and flexibility, non-biodegradable under normal environmental conditions, good chemical stability and biocompatible	[32–34]
chitosan (CS)	good film-forming property, soluble in acidified water, less flexible and have lower tensile strength, biodegradable and environmentally friendly and biocompatible	[35–37]

Despite the above-mentioned benefits of synthetic materials, there has been a significant contribution to developing naturally derived AEMs, further enhancing the environmental cleanliness of fuel cells as energy-generating devices. CS, cellulose, and their derivatives have emerged as promising alternatives owing to their low cost, abundance and biodegradability, making them more environmentally friendly [26,27,38–40], as they are low-cost and abundant materials that can fulfil the AEM’s requirements. Even though biopolymers have numerous advantages, they possess low mechanical strength and a higher swelling ratio, which adds instability to the overall integrity [41]. To address these limitations, chemical modifications, the introduction of fillers, and the preparation of blends with polymers exhibiting excellent stability and anion-exchange capacity compared to precursor materials have been employed.

This review aims to reveal the methods to enhance the CS polymer backbone for AEMs and overcome obstacles related to mechanical stability, ionic conductivity, water uptake, expansion rate and cell performance. There were several reviews published where CS was investigated as a proton-conducting membrane [42,43], and less as AEM. Among recent publications, Gorgieva’s team thoroughly reviewed CS’s chemical modifications and properties as a hydroxide transporter [14]. In addition to previously conducted studies, current work explores the latest innovations in CS AEMs. It makes an overview on the integration of pristine or functionalized CS with other polymers and inorganic additives. To get a comprehensive idea of the chemical modifications of CS, the simulations on the stability of cationic head groups grafted to CS were investigated. State-of-the-art cellulose membranes were discussed, as well as cellulose which is another suitable biopolymer for AFCs.

## 2. Chitosan-based anion exchange membranes

CS is the next most abundant polysaccharide on Earth after cellulose. It is derived from natural chitin by complete or partial N-deacetylation with the degree of deacetylation (DoD) usually varying between 70 and 95% [44]. CS is of great interest owing to its superior properties, such as biocompatibility, biodegradability, low cost and non-toxicity. Furthermore, strong hydrogen bonds can enhance water permeability, giving it good mechanical, film- or coating-forming properties [45–47]. The monomeric unit of CS, which contains an amine functional group, makes it cationic and hydrophilic, thus carrying hydroxide groups in alkaline conditions. CS membranes, while possessing several desirable properties, also have certain limitations in terms of conductivity, degradation and mechanical properties. CS, being a natural polysaccharide, is an insulating material and exhibits poor ion conductivity [48]. This can restrict its application in fields where ion conduction is essential, such as in the development of fuel cells. CS membranes generally possess relatively low mechanical strength and stiffness compared to synthetic polymers [49]. In some cases, the rapid degradation of CS membranes may limit their long-term stability and durability [50]. It is important to note that ongoing research and development efforts aim to address these limitations by exploring various strategies, such as the addition of conductive fillers, chemical modifications and composite material formulations. These approaches seek to enhance the ion conductivity, degradation rates and mechanical properties of CS membranes to broaden their range of applications.

Increased DoD [51] and high glass transition temperature [52] of CS facilitates the frailness of the formed film, and numerous research groups have reinforced CS membranes by integrating with organic or inorganic materials to compensate for this limitation. In the early research by Wan *et al.* [37], quaternized chitosan derivatives (QCS) were synthesized and cross-linked to form polymer membranes. In Nowacki *et al.* [53], CS was modified by glutaraldehyde (GA) in sodium hydroxide solution to improve the physico-chemical and electrochemical properties of the CS membrane applied in electric double layer capacitors.

The combination of CS with organic compounds is a promising area of research for use in AEM applications. In Hari Gopi *et al.* [54], cationically modified PVA and CS were cross-linked to get a gel polymer membrane for AEMs. In the paper of Yuan *et al.* [36], QCS was integrated with poly(diallyldimethylammonium chloride) (PDDA) to enhance the thermal and alkaline stability of the membrane; polysulfone was cross-linked with *N*,*N*-dimethyl chitosan to form a suppressed swelling and highly conductive polymer gel membrane, used in AEMs application [55].

Besides applying CS alone and fabricating CS together with organic materials, there has been growing interest in mixing CS with inorganic additives to prepare mixed matrix membranes. For example, in the research of Wen-chin Tsen’s group, QCS was combined with nanostructured fillers to enhance the stability and mechanical strength of the membrane (table 2). These include cationic silica-coated carbon nanotubes (CNTs) [40] or glycine betaine intercalated layered double hydroxides (LDHs) [56]. LDHs were dispersed uniformly within the membrane matrix, enabling efficient load transfer from the matrix to the stiff LDHs. This dispersion resulted in enhanced mechanical properties compared to a pure membrane. The tensile strength and elongation of the composite membrane with 5% intercalated LDHs content were reported to be 23.6 MPa and 51.4%, respectively. These values were 71% and 44% higher, respectively, than the corresponding properties of the pure membrane made of QCS and PVA. This indicated that the incorporation of intercalated LDHs significantly improves the membrane’s ability to withstand tension and deformation. Additionally, the LDHs and the intercalated QA groups contributed to enhanced ionic conductivity. These components acted as new OH^−^ conductive sites within the membrane. The composite membrane containing 5 wt.% intercalated LDHs demonstrated an ionic conductivity of approximately 35.7 mS cm^−1^ at 80°C, which is 42% higher than the pure membrane. Furthermore, the composite membrane exhibited improved alkaline stability. After immersion in a 1 M potassium hydroxide (KOH) solution for 168 h, the ionic conductivity of the composite membrane retained 70% of its initial value, whereas the pure membrane only detained 49% of its initial conductivity.

**Table 2.** The list of several recently reported composite membranes in AFCs.

polymer matrix	additives	methods	Ionic conductivity (mS cm^−1^)	major conclusions	ref.
chitosan (CS)	carbon nanotubes (CNTs)	*in situ* sol-gel coating	42.7 (80°C)	mechanical properties and electrochemical performance were improved	[40]
chitosan polyvinyl alcohol (CS/PVA)	layered double hydroxides (LDHs)	intercalation, incorporation	35.7 (80°C)	the thermal stability, tensile properties, and ionic conductivity were enhanced	[56]
chitosan (CS)	magnesium hydroxide [Mg(OH)_2_] graphene oxide (GO)	solution casting	142.5 (40°C)	mechanically stable with a high ionic conductivity	[57]
chitosan (CS)	poly[O-(2-imidazolyethylene) -N-picolyl (PIENP)	solution casting	10.2 (80°C)	chemical stability, ionic-exchange capacity and ionic conductivity were enhanced	[58]
polyvinyl alcohol (PVA)	molybdenum disulphide (MoS_2_)	solution casting	31.5 (RT)	the thermal stability and fire resistance were improved	[34]
polyvinyl alcohol (PVA)	bromomethylated poly(2,6-di-methyl-1,4-phenylene oxide) (BPPO)/MOFs	non-solvent induced phase separation	145.0 (80°C)	ionic conductivity and alkaline stability were improved	[59]
polyvinyl alcohol (PVA)	1-(4-formylbenzyl)-1-methyl piperidinium (FBMP)/TPA	solution casting	200.4 (80°C)	increased ionic conductivity and a high peak power density were obtained	[60]
polyvinyl alcohol (PVA)	poly(diallyldimethyl ammonium chloride) (PDDA)	solution casting	53.1 (RT)	tensile strength, water uptake and conductivity was enhanced	[61]

For the same purpose, Jiang *et al.* [34] prepared MoS_2_ filler with quaternized PVA through the solution casting method and used CS membrane as gel polymer for AEMs applications. The addition of MoS_2_ improved the mechanical strength of the composite membrane, probably owing to the reinforcing properties of the layered MoS_2_ material. Furthermore, the composite membrane exhibited a gradual reduction in methanol permeability with increasing MoS_2_ content. This characteristic is important in fuel cell applications since it helps to minimize methanol crossover and enhance overall efficiency. However, the IEC and ion conductivity of the QPVA/CS/MoS_2_ membranes initially increased with the addition of MoS_2_ but started to decrease beyond a MoS_2_ content of 0.2 wt.%. The QPVA/CS/MoS_2_ − 0.2 membrane achieved the highest ion conductivity value of 3.153 × 10^−2^ S cm^−1^.

Moreover, Kaker’s group combined CS with Mg(OH)_2_ and graphene oxide (GO) nanocomposite [57] and inorganic [40] materials. CS + Mg(OH)_2_ + GO + BTMAC AEMs exhibited improved hydroxide conductivity and low ethanol permeability, despite having a relatively high KOH uptake. The conductivity and ethanol permeability values reached were 142.5 ± 4.0 S cm^−1^ at a temperature of 40°C and 6.17 × 10^−7^ ± 1.17 × 10^−7^, respectively.

Ryu *et al.* [58] synthesized a novel membrane by copolymerizing CS with vinylimidazole derivatives, which achieved high hydroxyl-ion conductivity, excellent thermal stability and physical strength. At 80°C, the membrane demonstrated an enhanced hydroxyl ion conductance of 10.2 mS cm^−1^. The membrane also displayed a low water absorption ability of 39.49%, which indicates its ability to retain its structural integrity in the presence of moisture. Additionally, the membrane exhibited a low linear expansion ratio of 15.9%, despite its high ionic-exchange capacity of 1.57 mequivg^−1^. This suggests that the membrane is less susceptible to dimensional changes under varying conditions.

Various nanofillers are employed to make composite membranes, as illustrated in table 1. Adding nanoadditives to a polymer matrix can adjust membrane characteristics such as surface hydrophilicity, increase membrane stability in challenging conditions like high temperature and increase ionic conductivity compared to ordinary ion exchange membranes. By dispersing nanoparticles within the membrane matrix, they can act as reinforcing agents, making the membrane more resistant to physical stress, stretching, and wear and tear [62]. Papageorgiou *et al.* [63] investigated a substantial increase in materials’ hardness of reinforced poly-ether-ether-ketones with graphene nanoplatelets (PEEK/GNFs). When GNPs were introduced into PEEK, they acted as reinforcing agents and enhanced the material’s tensile properties. The introduction of GNPs also led to a linear increase in Young’s modulus with the concentration of GNPs [63]. In some cases, nanoadditives can increase the permeability of membranes. They can create nanoscale pores or channels that allow for faster transport of specific molecules or ions through the membrane while still blocking others [64]. The study conducted by Pandey *et al.* [65] reveals that introducing silver nanoparticles (AgNPs) into MXene nanosheets with varying loadings ranging from 0% to 35% can significantly increase permeability owing to the formation of new nanopores in the membrane by the attached silver nanoparticles. Nanoadditives can also enhance the selectivity of membranes. By carefully selecting and incorporating nanoparticles with specific surface properties, they can help tailor the membrane to selectively separate certain molecules or ions while excluding others. Additionally, nanoadditives can be used to control the structure and morphology of membranes. They can influence the size, shape and distribution of pores or other structural features, which can impact the membrane’s performance [66]. Abdollahi *et al.* [67] suggests that incorporating clay and bovine bone nanoadditives into ceramic nanocomposites can have a significant positive impact on the absorption of Ni (II) and Co (II) ions from wastewater [67]. This finding implies that these nanoadditives enhance the efficiency of ceramic nanocomposites as a material for wastewater treatment by increasing their ability to remove Ni and Co ions from the water with an efficiency of more than 95%. The choice of nanoadditives and their concentration must be carefully regulated, though, since an excessive amount might reduce the mechanical strength of the membrane and adversely impact its electrochemical characteristics [68].

### 2.1. Chemical modification

#### 2.1.1. Chitosan quaternization

**Figure 2.**
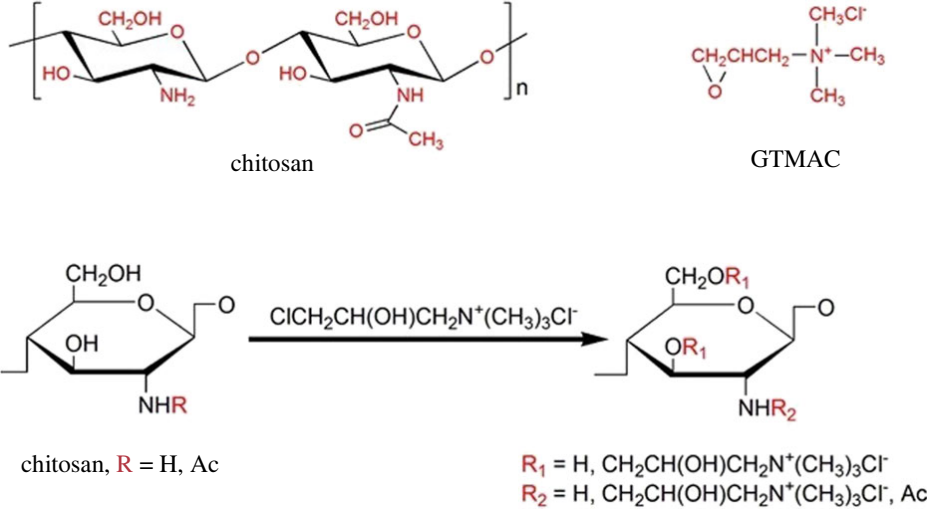
Chemical structure of CS, GTMAC and QCS.

Having free amino groups in the structure, CS can be efficiently functionalized (figure 2) [69]. Therefore, instead of using pristine CS, several research groups modified the CS material for AEM with various QA compounds, such as, glycidyltrimethylammonium chloride (GTMAC) [39,40,70,71], imidazolium group [20], hexadecyltrimethylammonium bromide [54] and 2,3,5-triphenyltetrazolium chloride [54].

Rao *et al.* [72] prepared AEM by polycondensation and functionalization with QA groups, which exhibited good chemical and thermal stabilities with high anion conductivity. As a result, contained hydrophilic and hydrophobic parts were separated, and imidazolium was chosen as a quaternary agent for the modification. Kim and his team [73] synthesized Im containing poly(ether-ether-ketone) denoted Im-PEEK. The introduction of bisphenol-A increased Im-PEEK’s solubility and imidazolium hydroxide moiety, enhancing water uptake, IC and IEC. The hydrophilic imidazolium group was controlled during the functionalization. Thus, the obtained membranes exhibited excellent conductivity under different temperatures. Ran *et al.* [74] reported a novel strategy for developing compatible imidazolium-based alkaline AEMs. According to the results, 1-methylimidazole was used to control IECs of membranes. In brief, imidazolium cations with conjugated structures displayed good thermal/chemical stabilities and solubility in polar solvents.

The amino groups of CS can be protonated in acidic pH, making it ionic and soluble by diminishing inter- and intra-molecular hydrogen bond networks between amine and hydroxyl groups [75]. The solubility of CS is reduced at neutral and basic pH ranges (above pH 6.5), thus limiting its applications. To overcome the low solubility of CS-specific free amino groups or hydroxyl groups, they can be chemically modified, e.g. by introducing positive charges into the polymer chain through quaternized agents [76]. According to literature, GTMAC [77], and 3-chloro-2-hydroxypropyltrimethyl ammonium chloride [78] are the most widely used for quaternary groups grafting (figure 2). Some studies revealed that polymers with various degrees of quaternization (DoQ) show different properties. In 2002, Hamman *et al.* [79] compared the absorption activity of peptide drugs in delivery. The quaternized CS derivatives with a DoQ of 22% showed increased absorption at pH = 7.4, and those with a DoQ of 50–60% were chosen as an optimum for enhanced permeation. Brasselet with colleagues [80] obtained CS derivatives with outstanding properties by using various grafting groups: N-carboxyalkyl [81]; O-carboxyalkyl [82–84]; N, O-carboxyalkyl [85,86]; phosphate [87]; and QA salts [88,89]. Thus, the presence of free amino and hydroxyl groups offers excellent opportunities for the modification of CS.

The functional group that may transport the hydroxyl anions (i.e. cationic groups harbouring a hydroxyl counter-anion) and the backbone’s composition significantly impact the membrane’s chemical and thermal durability. Compared to quaternary phosphonium and tertiary sulfonium groups, QA groups are more chemically and thermally stable. Additionally, a cross-linking procedure can also increase the stability of the membrane [7].

#### 2.1.2. Chemical cross-linking

During the quaternization process of CS, the primary polymer backbone stays unchanged. Therefore, CS chains are unfolded by electrostatic repulsions from the increased cationic groups on CS, leading to improved solubility [90]. On the other hand, polymers with a high DoQ can enhance hydrophilicity and lower membrane strength. To overcome these problems, highly-stable cross-linked AEMs by various agents have been prepared [91,92].

**Figure 3.**
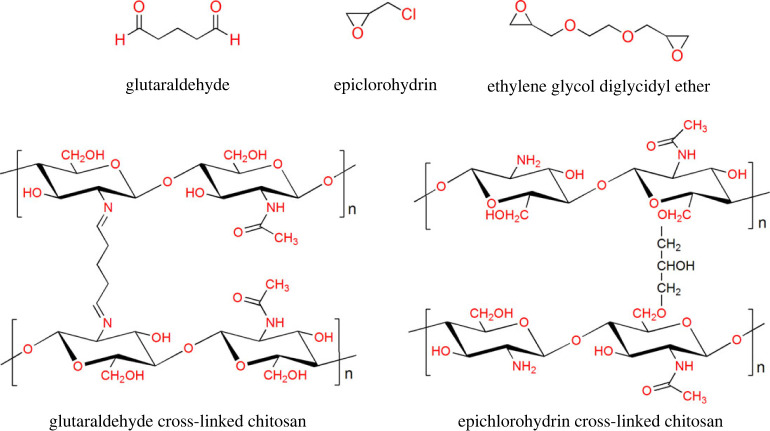
Chemical structures of cross-linking agents and cross-linked CS polymers.

Cross-linking of CS has been conducted by different substances (figure 3), mainly on free -NH_2_ and -OH groups, similarly to other modification types. For instance, cross-linking of CS with GA occurs between aldehyde and -NH_2_ groups. However, cross-linking with epoxide groups epichlorohydrin, trimethylolpropane triglycidyl, and ethylene glycol diglycidyl ether occurs on -OH sites of CS [39,56,93].

Wan *et al.* [94] have investigated quaternized CS’s potential for use in AEMs. After being derived, *N*-[(2-hydroxy-3-trimethylammonium)propyl] CS chloride was cross-linked to produce membranes with IECs and conductivities that ranged from 0.33 to 0.86 meq g^−1^ and from 4.8 up to around 8.0 × 10^−3^ S cm^−1^, respectively.

The nature of the cross-linker is an essential factor in the conductivity of AEMs. Introducing more ion exchange groups leads to membrane swelling [95]. Thus, QA groups can be coordinated by water molecules, which transport OH^−^ anions and affect ion exchange capacity. Hydrophilic and hydrophobic cross-linked membranes have demonstrated a high (20%) and low (5%) water uptake and swelling ratio, respectively. As a result, membranes modified by hydrophobic cross-linking have shown a reduction in OH^−^ conductivity and IEC [27].

### 2.2. Chitosan-based interpenetrating polymer networks

As discussed above, cross-linking is implemented to control the dimensional swelling of membranes. Considering the shortcomings of this modification, including inefficient ionic conductivity or weak mechanical integrity of the membranes [96–98], membranes could be fabricated using an IPN. Two or more polymers are at least partly or wholly entangled but have no chemical bond in between [98,99]. This method boosts the material’s durability and strength while keeping each component’s unique advantages. Besides fully cross-linked IPNs, there are pseudo- or semi-IPNs, where both linear chains of one polymer and cross-linked chains of the other are present. Thus, two polymers are interlaced only to some extent [99,100]. The whole entanglement is possible by introducing cross-linking agents [96].

Since IPNs are crosslinked, they swell but do not disintegrate in the presence of solvents, and in addition, creep and flow may be almost completely inhibited. As a result, the material’s physical and chemical properties—such as temperature sensitivity and interfacial compatibility, are improved while its mechanical strength and elasticity increase. The constituent polymers’ characteristics influence their characteristics and how they are mixed [99].

Such systems can be fabricated by sequential and simultaneous polymerization [99,101]. In the sequential method, the polymerization of one monomer occurs in the presence of another polymer, and two polymers are cross-linked afterwards. However, the fabrication of simultaneous IPNs differs from the previous one as this type involves blending two prepolymers and their separate cross-linking.

CS-based IPN structures were widely investigated for pharmaceutical and biomedical applications. For example, there are reported cases of integrating CS with natural polymers, including alginate for drug delivery [102], and gelatin for tissue engineering [103]. In addition, synthetic polymers such as polyvinylpyrrolidone, polyacrylic acid, polyacrylamide, polyethylene glycol and polyacrylonitrile were selected for CS-based IPN hydrogel preparation [102].

For the fabrication of anion conducting IPN with decent dimensional stability, CS was combined with polystyrene (PS) [100], poly (acrylamide-co-diallyl dimethylammonium chloride) (PAADDA) [104,105] and block copolymers polystyrene/polyacrylamide (PS/PAM) [96]. In all cases, the CS content was above 50 wt.% in the composition of membranes.

Additional cross-linking of polymer chains through thermal or chemical treatment can be done to prevent undesirable phase separation. For example, some studies introduced GA as a final step of membrane preparation [96,105], and others used both types of cross-linking [104].

The hydroxide transport capability of such CS-based IPN membranes was up to 4 × 10^−2^ S cm^−1^ at 80°C [96,100,104,105]. The tensile strength was generally above 20 MPa and significantly depended on the nature and content of other polymers. For example, the tensile strength of CS-based IPN AEM drops from 31.4 MPa to 23.7 MPa with the increase of hydrophilic PAADDA’s concentration from 33 to 43% [104]. However, when the content of copolymers PS/PAM rose in the same amount, the membrane strength slightly improved from 41.4 to 43.9 MPa.

Generally, the choice of fabrication method can be as crucial as the AEM’s composition for reaching decent mechanical stability.

### 2.3. Chitosan-based composite membranes

The preparation of composite membranes is another way of supplying ion transfer sites and restraining membrane swelling by enhancing mechanical properties. Composite or hybrid membranes comprise two or more materials. For CS-based AEMs, CS works as the matrix phase, and fillers (organic and inorganic types) are the dispersed phase. Many typical nanofillers applied in such membranes include zero-dimensional materials (SiO_2_ [26,28,106,107] and tetrabutyl titanate [108] particles), one-dimensional materials (CNT [39,40] and halloysite nanotube [109]), or advanced two-dimensional materials (GO [57,110–113], montmorillonite [114,115] and MXene [15]).

It is worth mentioning that not all inorganic fillers have a chemical affinity to organic material as CS. Therefore, one should consider addressing this question too, when developing hybrid membranes. For example, Shi and co-workers ensured strong bonding between SiO_2_ and CS by tuning inorganic nanoparticles with imidazolium groups [26].

#### 2.3.1. Carbon nanotubes

Having excellent physical, electrical and chemical properties, carbon materials, especially CNTs, were extensively studied as electrodes used in energy science for energy conversion and storage, including fuel cells [116–119], nano-sensors [120], solar cells [41,121] and supercapacitors [122]. CNTs are tubes rolled up as a hollow cylinder shape by one atom thickness of graphene sheets, typically measured in nano-metres [123,124]. Also, by the number of an array of graphene sheets, CNTs could be classified as single-wall carbon nanotubes (SWCNT), formed by a single layer of graphene sheet or multiwall carbon nanotubes (MWCNT), developed by two or more layers of graphene sheets.

**Figure 4.**
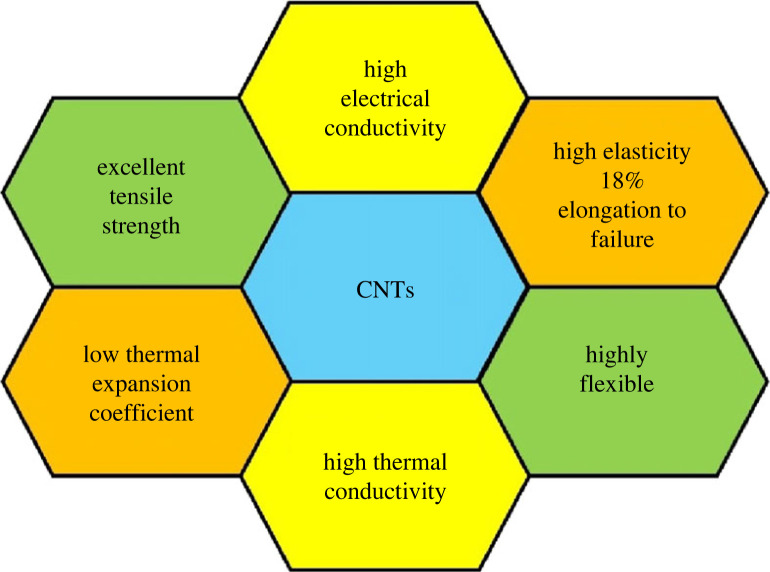
Schematic diagram of an SWCNT, MWCNT and properties of CNTs.

CNTs are assured of their stunning conductivity properties, which could be simplified as shown in figure 4.

**Figure 5.**
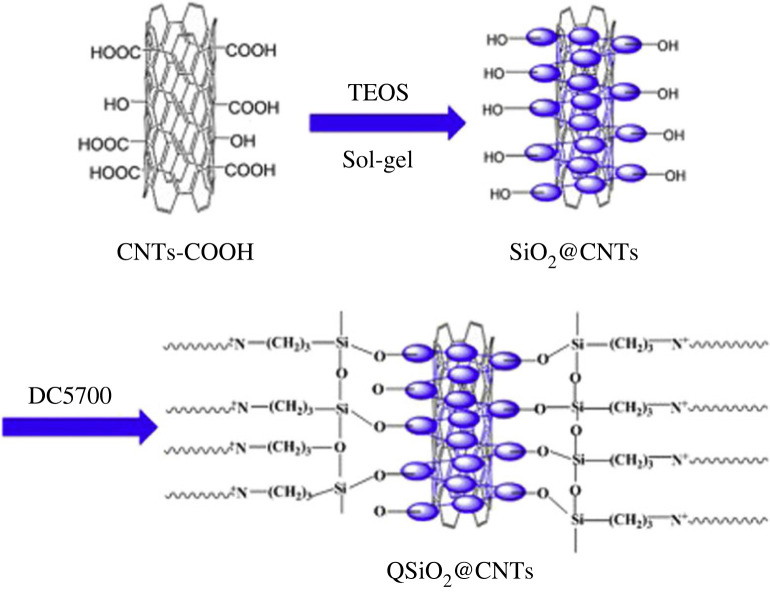
Schematic illustration of the preparation of QSiO_2_@CNTs. Retrieved from [40].

Using the sol-gel method, Jang and his team [40] fabricated QCS AEMs with functionalized CNTs. To prepare functionalized CNTs, QSiO_2_@CNT, CNTs were coated with a thick layer of silica and then quaternized with an ammonium group figure 5. Comparing results, QCS membranes with $5\;\mathrm{wt}.\%\;{\mathrm{QSiO}}_{2}@\mathrm{CNT}$ obtained 42.7 mS cm^−1^ ionic conductivity at 80°C, nearly three times that of QCS membranes. As mentioned above, the quaternization provided perfect interfacial cooperation between QSiO_2_@CNT and QCS, the homogeneous dispersion of QSiO_2_@CNT inside QCS membranes, and the existence of an extra hydroxide ion channel within the composite membranes. It drives the improvement of mechanical performance as well as electrochemical properties.

**Figure 6.**
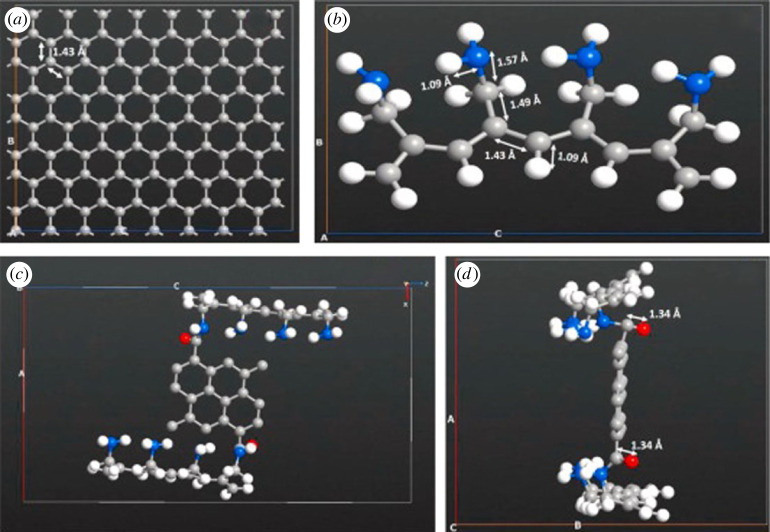
Modelled structure representation of (*a*) graphene, (*b*) poly allylamine (PAA) and (*c*,*d*) PAA-GO covalently attached component [128].

Besides CNTs, GO is another carbon-based material studied extensively as nanofillers in fuel cells [125–127]. Unlike a CNT, GO has hydrophilic and hydrophobic characteristics. GO shows high hydrophilicity, attributed to the oxygenic functional groups—epoxy, hydroxyl and carboxylic—attached to its carbon atoms (figure 6). This property could help transfer anions across the polymer electrolyte when GO is used as nanofillers in AEMs. Moreover, the aromatic ring (sp^2^ carbon layer) on GO provides a robust covalent bonding to enhance the mechanical strength of AEMs [57,116,129,130].

In the research work of Yang *et al.* [111], GO was added as nanofillers into PVA/CS mixed polymer electrolyte. The research found with the increase of GO content, the swelling ratio for various nanocomposite membranes decreased, and tensile strength was enhanced. More commonly than using GO alone as a nanofiller, more research has been focused on functionalized GO recently to improve composite membrane performance as a whole significantly. Chen *et al.* [112] reported novel nanohybrid CS AEMs used imidazolium-functionalized graphene oxide (ImGO) as a nanofiller. The AEMs with ImGO obtained excellent thermal, mechanical and anti-swelling stabilities. The ligands on QA groups on ImGO help construct hydroxide ion transport highways at CS/ImGO interface via interfacial interactions, resulting in OH conductivity as high as 102 mS cm^−1^ at 90°C, nearly four times that of pristine CS membranes [112]. Božič and co-workers [110] combined Mg(OH)_2_, GO with benzyltrimethylammonium chloride nanofillers and CS polymer electrolyte, and generated thermally and mechanically stable AEMs with the ionic conductivity of 142 mS cm^-1^ at 40°C.

#### 2.3.2. MXenes

Besides carbon-based fillers, there is growing interest in introducing a class of synthetic additives such as MXenes. As was mentioned above, MXenes are two-dimensional materials, which comprise early TM carbides and carbonitrides, including titanium carbide, titanium carbonitride, niobium carbide and vanadium carbide with the general formula M_*n*+1_ X_*n*_ [131,132]. They are derived from MAX phases (M_*n*+1_AX_*n*_), where ‘M’ stands for early TM, ‘A’ is the element from groups 13 and 14 of the periodic table, and ‘X’ - C and/or N. The most promising MXene fabrication technique is the exfoliation of ‘A’ sheets from the structure of the precursive MAX phase in a harsh environment. So far, scientists have investigated chemical etching using hydrofluoric acid (HF) or fluoride salts treatment or electrochemical etching using HCl [133]. Considering the toxicity and corrosiveness of HF, other etchants, such as concentrated NaOH and tetramethylammonium hydroxide (TMAOH), have been suggested, too [134]. The efficiency of the etching method depends on the nature of MXene, e.g. titanium carbide was more electrochemically effective as a supercapacitor when had been exfoliated by lithium fluoride dissolved in hydrochloric acid than traditional etchant-HF [131].

**Figure 7.**
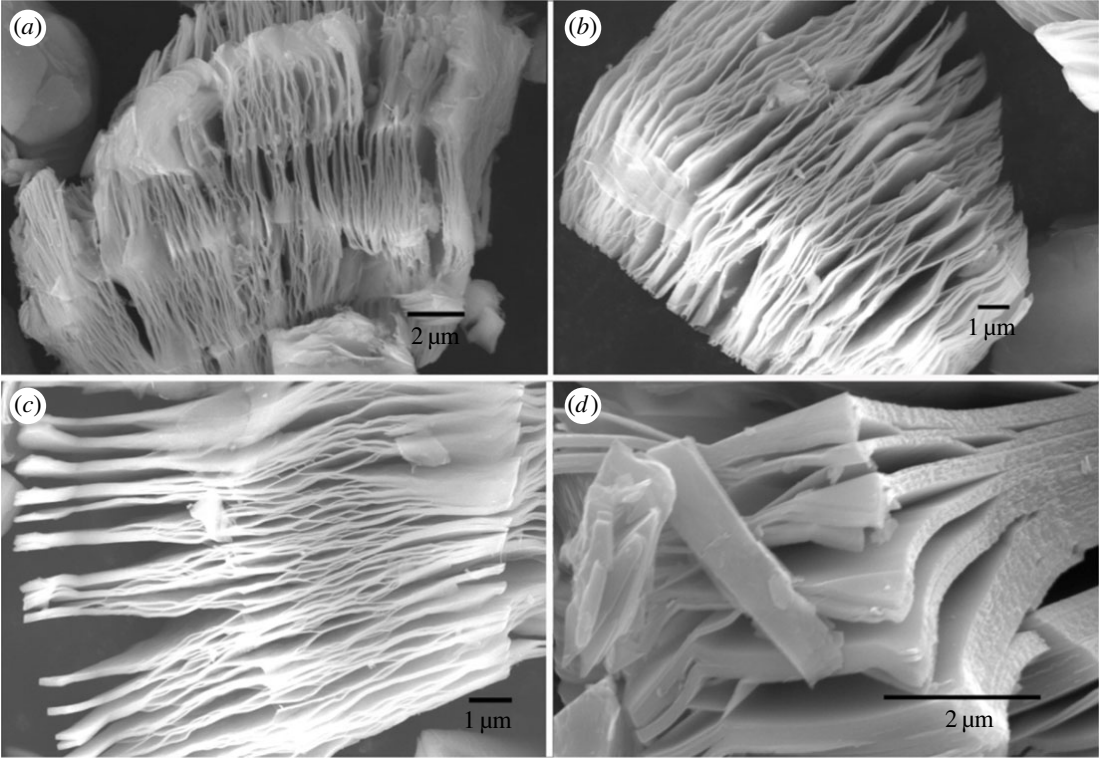
Scanning electron micrograph of Ti2C layers exfoliated by sodium fluoride. Retrieved from [131].

The surface morphology of all members of the MXene family is similar to each other and resembles the layered structure of graphite [131]. For example, figure 7 depicts the side view of titanium carbide obtained from the precursor by exposure to fluoride salt. Despite the external similarities, the properties of MXenes are more readily adapted to their applications’ requirements than graphite [133].

Since their discovery, MXenes have been widely applied in energy storage and generation systems owing to their desirable characteristics, including high electron conductivity for electrode materials and photocatalysts [133,134], dispersibility and enhanced ion conduction, thermal and tensile properties for polymer electrolyte membranes in fuel cells [135–138].

A vivid example of improved ionic conductivity is the development of composite CS/functionalized MXene AEM. Wang and Shi [15] obtained thermally resistant and mechanically strong membranes by blending quaternized MXene (titanium carbide) with CS in different percentage content (2.5, 5, 7.5 and 10 wt.%). The hydroxide transfer of the hybrid AEM was almost three times greater than that of pristine CS owing to the creation of continuous channels by MXene sheets. The addition of QMXene-NH_2_ to a hybrid membrane enhanced the thermal and mechanical stability of the membrane. Interestingly, despite the improved ion exchange capacity, the water uptake ratio of the hybrid membrane decreased with the incorporation of QMXene-NH_2_. As a result, the membrane became less prone to excessive water absorption, which helped to maintain its stability and performance. Furthermore, the swelling ratio of the hybrid membrane also decreased upon the addition of QMXene-NH_2_. To provide a specific example, when 7.5% QMXene-NH_2_ was incorporated into the hybrid membrane, the ion exchange capacity increased to 85.4% compared to the base membrane without QMXene-NH_2_. Simultaneously, the water uptake ratio dropped to 18.4% compared to the base membrane. These numbers illustrate the significant impact of QMXene-NH_2_ on the hybrid membrane’s properties.

#### 2.3.3. Layered double hydroxides

**Figure 8.**
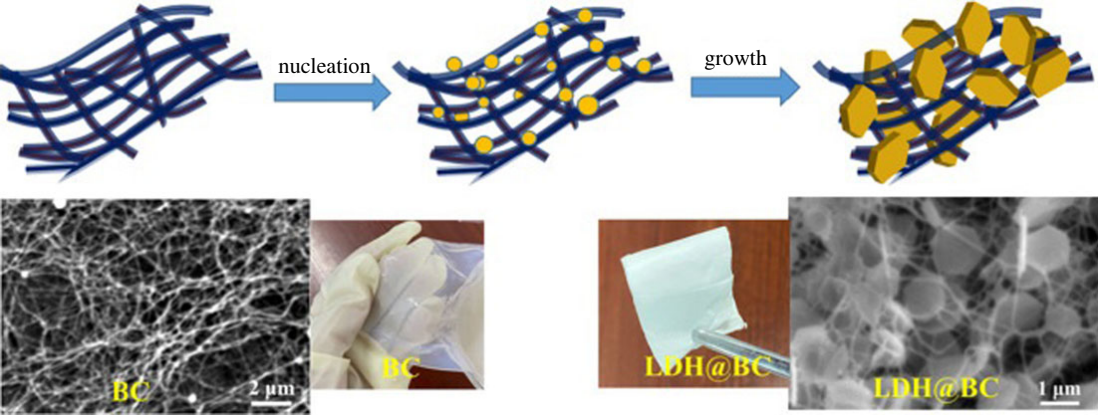
Schematic illustration of the preparation of LDH@BC. Retrieved from [140].

LDHs have recently been shown to be superior inorganic anionic conductors. Owing to the positively charged nature of LDHs, the interlayer’s linked water molecules and the host layers’ plentiful hydroxyls work together to build dense hydrogen bond networks over the two-dimensional surface, which makes it easier for OH ions to diffuse through the surface [139]. Ni *et al.* [140] reported a quick and eco-friendly method to successfully create LDH@BC (bacterial cellulose) bifunctional porous substrates simultaneously acting as ion transport media and solid reinforcing substrates figure 8.

Water in AEMs serves as a medium that facilitates the dissociation of OH^−^ ions and provides pathways for ion conduction via hydrogen bond networks. This phenomenon is critical for the efficient operation of various electrochemical devices that rely on AEMs for ion transport. However, excess water can lead to severe solvent swelling in AEMs, which in turn can pose significant challenges to both the dimensional and mechanical stabilities of the membranes. Moreover, if excessive water uptake occurs, it can hinder the movement of ions and subsequently decrease the overall conductivity of the system. Therefore, controlling and maintaining the water content within the AEM through proper hydration and humidification strategies can help strike a balance between ensuring sufficient ionic conductivity and avoiding excessive swelling. Based on the various functional groups and nanomaterials found in QCS-based AEM, table 3 lists water uptake, swelling ratio and IEC results of some AEMs that are suitable for AEMFCs.

**Table 3.** Properties of QCS composite membranes suitable for AEMFCs.

membrane	water uptake (%)	swelling ratio (%)	ion-exchange capacity (mmol g^−1^)	ref.
QCS/QSiO2@PVDF	150 (80°C)	<50 (80°C)	n.a.	[28]
QCS/0.15-LDH@BC	142.1 (80°C)	67.5 (80°C)	1.97	[140]
QCS/PVA-6% LDH	243 (80°C)	125	2.54	[141]
QCS/PVA-1%-LDH@CNTs	133 (80°C)	103 (80°C)	1.65	[39]
QCS/PVA-5%-B-LDH	≈170 (80°C)	≈95 (80°C)	n.a.	[56]
(PU/HPW/QCS/HPW)200	12.3 (80°C)	7.7 (80°C)	1.33	[142]

Liu *et al.* [28] increased the overall hydrophobicity of the QCS-based membrane by incorporating PVDF nanofibres into the composite. As a result, the membrane has become less prone to absorbing water, thereby decreasing its swelling ratio to less than 50%. This is because, nanofibres have a high surface area to volume ratio, and their presence can create a network of barriers that restrict the penetration of liquids into the membrane matrix. This can further contribute to the reduction of swelling.

The IEC is a measure of the number of ion exchangeable groups (usually functional groups like QA or other anion-exchangeable sites) present in the membrane material. The higher IEC value in an AEM is generally indicative of better ion transport properties and can lead to improved ionic conductivity and electrochemical performance. According to the IEC results in table 3, the increasing of three-dimensional LDHs concentration in the composite membrane leads to a higher IEC, and the composite membrane with 6% LDHs has the highest IEC value among the other compositions. This phenomenon can be explained in terms of two factors: (i) because of the presence of exchangeable charge compensating anions in the interlayer regions, LDHs exhibit excellent ion exchange capacity. This means that LDHs can readily exchange the anions in their interlayers with other anions from the surrounding solution [143]; and (ii) since the composite membrane is formed using a blend of QCS and PVA, the crystalline degree of this blend matrix is reduced. As a result of this reduction in crystallinity, the composite membrane gains the ability to promote the dissociation of anions.

LDH laminates were successfully prevented from layering up with the help of the BC nanofibre template, leaving their anion-conductive sites fully exposed for quick hydroxide ion transport through the composite membranes. Additionally, the three-dimensional porous substrate created by combining BC nanofibres with LDHs significantly increased the mechanical strength of the pore-filled QCS polymer [140]. Compared to the QCS/BC and pure QCS membranes, the tensile strength of the QCS/0.25-LDH@BC increased by 83.7% and 250%, respectively, owing to the reinforcement effect of the LDH@BC porous substrate (table 4).

**Table 4.** Main characteristics of different layered anion exchange membranes.

membrane	ionic conductivity (mS cm^−1^)	alkaline stability (%, residual ionic conductivity)	mechanical strength (MPa)	power density (mW cm^−2^)	ref.
QCS/PS-semi-IPN	28.0 (80°C)	95% (1M KOH, RT, 72 h and 60°C 50 h)	20.0	n.a.	[100]
CS/PAADDA/PUB	31.2 (80°C)	93% (1M KOH, RT, 48 h)	22.7	38.1 (80°C)	[105]
QCS/0.15-LDH@BC	42.5 (80°C)	86% (2M KOH, 30°C, 200 h)	64.3	84.2 (60°C)	[140]
QCS/PVA-6% LDH	25.7 (80°C)	92% (2M KOH, 25°C, 100 h)	26.6	73 (60°C)	[141]
QCS/PVA-1%-LDH@CNTs	47.0 (80°C)	65% (1M KOH, 40°C, 100 h)	39.9	107.2 (80°C)	[39]
QCS/PVA-5%-B-LDH	35.7 (80°C)	70% (1M KOH, RT, 168 h)	23.6	97.8 (60°C)	[56]
(PU/HPW/QCS/HPW)200	49.1 (80°C)	65% (1M KOH, 80°C, 888 h)	9.4	n.a.	[142]

When compared with QCS/BC, the composite membranes’ anion conductivity increased by 52.5% thanks to LDHs’ capacity for anion transport. Zhao *et al.* [141] created and analysed multiple QCS/PVA-LDH composite membranes with varying three-dimensional LDH content. Owing to the addition of evenly distributed three-dimensional LDHs, which can act as physical cross-linking points to prevent polymer chain rupture, the mechanical strength of the composite membranes was enhanced. The QCS/PVA-6% LDH composite membrane, as a result, experienced a 46% increase in ionic conductivity, and after 100 h of immersion in a KOH solution, its residual conductivity could still be 92%. The composite electrolyte’s maximum power density increased, showing a value of 73 mW cm^−2^, as opposed to the pristine membrane’s value of 40 mW cm^−2^, owing to the enhanced ionic conductivity and decreased methanol permeability [141].

*In situ* co-precipitation with NH4F was used to create a hierarchical nanostructure comprised of one-dimensional CNTs and two-dimensional LDH laminates, which were subsequently included in the QCS/PVA mix matrix to make composite membranes. Compared to the pure QCS/PVA membrane, the resulting composite membranes exhibit noticeably higher mechanical strength and ionic conductivity [39]. Gong *et al*. showed that the QCS/PVA-LDH@CNTs composite membranes are a potential AEM option owing to the most excellent ionic conductivity of 47 mS cm^−1^ at 80°C 61% greater than that of the pure membrane (only 29 mS cm^−1^). As a result, while employing 2 M methanol+5 M KOH as the anode fuel at 80°C, QCS/PVA-1%-LDH@CNTs electrolyte produces an open circuit voltage of 0.83 V and a peak power density of 107.2 mW cm^−2^, whereas for pure membrane these two values showed only 0.79 V and 84.2 mW cm^−2^, respectively. The QCS/PVA mixed matrix containing glycine betaine-intercalated hydrotalcite provides additional ion transfer channels at the interlayer and surface, which significantly enhances ionic conductivity performance [56]. The QCS/PVA-5%-B-LDH membrane exhibited a peak power density of 97.8 mW cm^−2^ and an ionic conductivity of 35.7 mS cm^−1^. Furthermore, B-LDH played a crucial part in the physical cross-linking point, resulting in the creation of composite membranes that were more compact and durable. Even after being submerged in 1 M of KOH solution for 168 h, the composite membrane’s alkali stability was high. The QCS/PVA membrane’s ionic conductivity drops by half, whereas the QCS/PVA-5%-B-LDH membrane maintains a 70% value.

## 3. Cellulose-based anion exchange membranes

CS and cellulose are both biopolymers that have been investigated for their potential use in AEMFCs. CS has been shown to have good ion conductivity and stability in alkaline conditions, which makes it a promising material for use in AEMFCs. However, one of the challenges of using CS is its relatively high cost compared to other materials. Cellulose, on the other hand, is the most abundant biopolymer on Earth and is derived from plant matter. It has also been studied as a potential material for AEMFCs owing to its high chemical stability and low cost. Cellulose is considered one of the widely commercialized biopolymers, as it is the most abundant biosynthetic product from plants, animals and bacteria [144]. It is also well known for its biodegradability and bio-renewability, which perfectly contributes to using alternative energy sources. Cellulose has a suitable polymer backbone for AEM application owing to its low density, low weight, excellent mechanical properties, low cost and possibility for different functionalization owing to the plenty of OH^−^ groups on its structure [145].

Different chemical properties and production methods distinguish the derivatives of cellulose, and a number of them have been studied as potential AEM, including nanocrystalline cellulose (CNC), nanofibrillar cellulose (CNF), BC and cellulose acetate. Derivatives mentioned above have their own advantages as well as disadvantages. For instance, CNFs have a high surface area and interchanging crystalline structure, while BCs are known for their ultra-high crystallinity and polymerization degree, and it is also free of lignin [146,147].

Regarding the challenges of using cellulose and its derivatives in AEM application, the main one is the low conductivity of the cellulose and its high swelling ratio, which affects the overall performance of the membrane in a real fuel cell system. To address this challenge, researchers have explored various strategies to enhance the conductivity of cellulose-based AEMs. In particular, referring to the conductivity problem of the cellulose-based membrane, there are numerous types of agents to improve the conductivity of AEM, including PDDA [148], trimethylaluminium [149], 2,2-azobis(2-methylpropionitril) and N-bromosuccinimide [150]. Compared to others, DABCO (1,4-diazabicyclo [2.2.2]) is the most promising quaternary agent [43]. Gautam das *et al.* quaternized cellulose with DABCO (1,4-diazabicyclo[2.2.2])octane and cross-linked it with polysulfone backbone, achieving 74.23 mS cm^−1^ high conductivity moreover, the membrane showed good dimensional and alkaline stability owing to cross-linking with compatible polymer [38]. Another promising approach to improve the ionic conductivity of the bio-based AEM was conducted by Zou *et al.* [148], where BC membrane was used as a polymer matrix for the electrochemical reduction of CO_2_. The nanoporous structure of the membrane was obtained by the reaction between hydroxyl groups of BC with the aldehyde group of GA, thus ‘trapping’ the PDDA into the polymer skeleton. This mechanism showed ionic conductivity at 38.98 mS cm^−1^ at 80°C [148].

Reactive hydroxyl groups of cellulose make it compatible with several polymers and organic [144] and inorganic compounds [55,151] and the fabrication of hybrid membranes with synthetic polymers [38,152] is also considered an effective method to improve the IEC of the membrane, hence, the IC.

Blending cellulose with synthetic polymers can be considered a reassuring method of fabricating bio-based AEM, just as for CS, because both have insufficient mechanical properties owing to excessive water absorption [12]. For example, Lu *et al.* [27,152] synthesized a CNC-based composite membrane cross-linked with PVA, which showed a low swelling ratio and slightly higher ionic conductivity (0.044 and 0.053 S cm^−1^) compared to commercially available FAA AEM owing to using hydrophobic binder between two polymers.

The study by Cheng *et al.* focused on incorporating quaternized nanocrystalline cellulose (QCNC) into a polymer matrix of quaternized poly(phenylene oxide) (QPPO) to improve the properties of AEMs [150,153]. They varied the proportion of QCNC and QPPO in order to enhance the overall properties of the AEM (table 5). One of the properties they investigated was the OH^−^ ion conductivity, which is an important factor for AEM performance. By adding QCNC to the QPPO membrane, a significant improvement in OH^−^ ion conductivity was achieved. The reported ion conductivities for different membrane compositions were as follows: QPPO: 16.7 ± 0.2 mS cm^−1^, QPPO/QCNC-0.5: 19.3 ± 0.6 mS cm^−^^1^, QPPO/QCNC-1: 21.3 ± 0.6 mS cm^−1^, QPPO/QCNC-2: 28.0 ± 0.1 mS cm^−1^, QPPO/QCNC-3: 20.5 ± 0.3 mS cm^−1^, QPPO/QCNC-4: 13.9 ± 0.7 mS cm^−1^. Among the different QCNC loadings, the QPPO/QCNC-2 membrane exhibited the highest ion conductivity. This improved performance was primarily attributed to the efficient dispersion of QCNC within the QPPO polymer matrix. The homogeneously distributed QCNC created excellent hydrophilic channels within the QPPO membrane, allowing for effective adsorption of water molecules and promotion of OH^−^ ion transport. Furthermore, the QPPO/QCNC-2 membrane demonstrated moderate alkaline stability at 80°C in a 1 mol l^−1^ NaOH solution during long-term measurements. Even after 120 h, the membrane possessed ion conductivity of 5.0 mS cm^−1^, QPPO/QCNC-2 showed a more significant cell performance, as shown in table 5. As can be seen, power density of QPPO/QCNC-2 reached 392 mW cm^−2^ compared to pure QPPO with a value of 270 mW cm^−2^ at 60°C.

**Table 5.** Quaternized cellulose-containing membrane characteristics and performance outcomes.

membrane	water uptake (%)	swelling ratio (%)	ion-exchange capacity (meq g^−1^)	ionic conductivity (mS cm^−1^)	mechanical strength (MPa)	power density (mW cm^−2^)	ref.
QCNC/QPPO	15.1	2.2	1.00	16.7 (20°C)	28.3	270 (60°C)	[150]
QCNC/QPPO-0.5 wt.%	18.0	2.4	1.01	19.3 (20°C)	28.9	n.a.	
QCNC/QPPO-1 wt.%	17.7	2.2	1.06	21.3 (20°C)	28.6	n.a.	
QCNC/QPPO-2 wt.%	16.9	2.6	1.05	28.0 (20°C)	30.9	392 (60°C)	
QCNC/QPPO-3 wt.%	16.8	2.4	1.00	20.5 (20°C)	22.8	n.a.	
QCNC/QPPO-4 wt.%	17.6	2.3	1.04	13.9 (20°C)	20.2	n.a.	

The combination of quaternized cellulose fibre (QCF) and quaternized graphene oxide (QGO) with QPPO by crosslinking suggests a multi-functional approach [55]. Their results suggested that incorporating QCF and QGO fillers into the QPPO matrix has a significant impact on ion exchange capacity and ion conductivity. The composition with a ratio of 100 : 1 : 1 (QPPO : QCF : QGO) appeared to be the most effective in terms of both ion-exchange capacity and ion conductivity, with an IEC of 2.64 meq g^−1^ and an ion conductivity of 114.64 mS cm^−1^ at 25°C. These findings suggested that incorporating quaternized cellulose nanocrystals (QCNC) into the QPPO polymer matrix can significantly enhance the OH^−^ ion conductivity and overall performance of AEMs for potential applications in various fields.

Therefore, optimizing the structure or cellulose content in polymer membranes can have a profound impact on their properties, making them more suitable for fuel cell application. These improvements can enhance their ability to handle water, exchange ions, conduct ions, and maintain stability under different conditions, ultimately leading to better performance in relevant applications.

## 4. Computational studies in anion exchange membrane fuel cells

Computational studies play an essential role in exploring the chemical stability of QA head groups and hydroxide (OH^−^) ion transportation in AEMFCs. Particularly, coarse-grained molecular dynamics (MD) simulations are commonly implemented to understand the microphase-segregation morphology and transportation of OH^−^ ion for PEEK, PPO, PS and PS-based AEM in the presence of explicit water [154–161]. However, there were no conducted coarse-grained MD works for the biopolymer-based backbone of AEMFCs.

**Figure 9.**
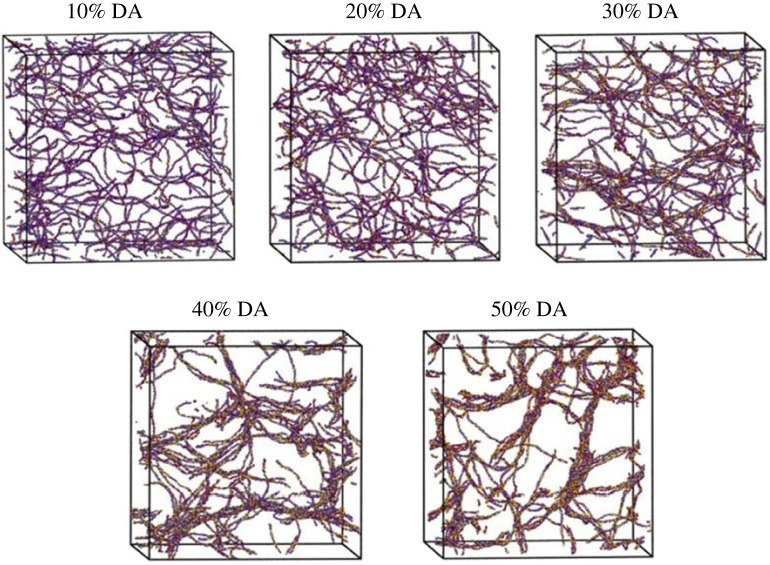
Snapshots at the end of coarse-grained MD simulations of 1.5 wt.% chitosan solution with degree of acetylation (DA) from 10% to 50% [162].

At the same time, there are many coarse-grained MD models of biopolymers such as CS, chitosan composites, chitin, cellulose and other types of biopolymers for various applications. For instance, Benner & Hall [162] developed a coarse-grained model for CS of any molecular weight, degree of acetylation (DA) and CS concentration to predict the solution behaviour as shown in figure 9. The results of coarse-grained MD simulations reveal increased self-assembly of CS molecule in solution with increasing DA and concentration of CS [162].

**Figure 10.**
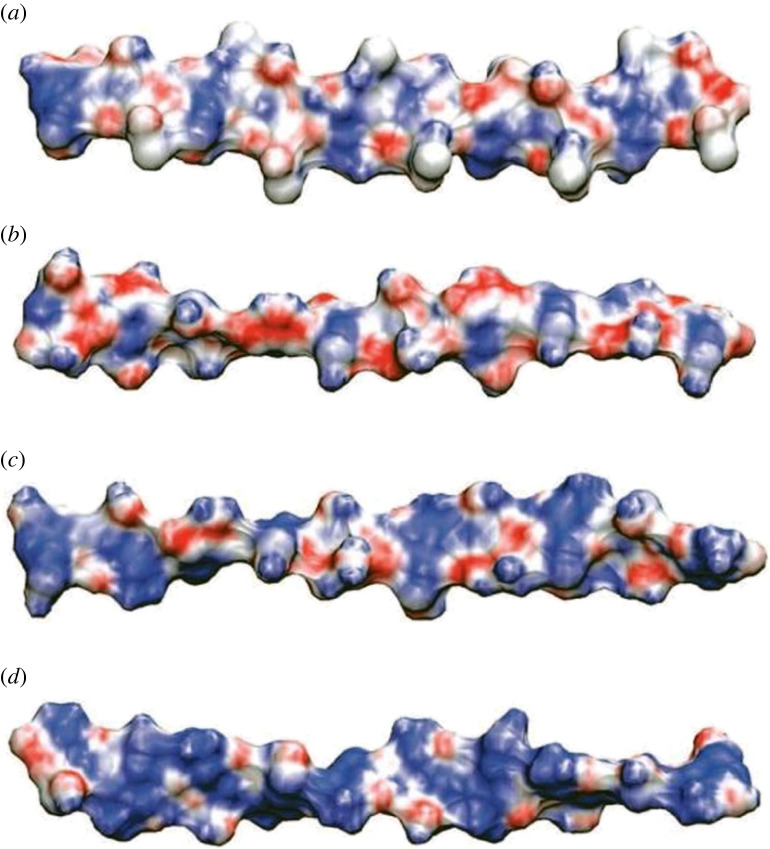
Molecular electrostatic potential of (*a*) chitin; (*b*) chitosan at high pH; (*c*) chitosan at neutral pH, and (*d*) chitosan at low pH [188].

In addition, the nanophase-segregated structure and transportation of OH^−^ ion via QA head group of PPO, polyvinyl benzyl, polynorbornene, PS, PSF and PEEK, polyarylene (ether sulfone ketone) based AEM have been well studied by reactive and classical all-atom MDs [163–176]. Moreover, many density functional theory (DFT) calculations study the transition states, activation energy and reaction energy of degradation pathways for various QA head groups of AEMs [168,177–186]. However, there were no investigated DFT and MD works for biopolymeric backbone-based AEM. Meanwhile, there are conducted DFT calculations and MD simulations to study the structural properties of monomers of biopolymers for various applications. For example, Franca *et al.* [187] studied the structural properties of chitin and CS chains in aqueous solutions via MD simulations and DFT calculations. The results show that chitin adopts the twofold helix, while CS adopts five distinct helical motifs and its conformational equilibrium is highly dependent on pH as can be seen in figure 10. Also, it is found that the molecular surfaces of chitin and CS at high pH exhibit a similar electrostatic profile. Computational modelling and simulation should be advantageous in predicting the QA stability and transportation of OH^−^ ion in biopolymer-based AEM. Table 6 demonstrates the various modelling methods that may be used for the characteristics of AEMs used in AFCs. Additionally, it also illustrates how various modelling length scales have been applied to investigate the AEM qualities in earlier studies.

**Table 6.** The list of different modelling studies on AEMs.

method	tools	ref.	explored properties
*ab initio* calculation	DFT	[178,184,189]	degradation in alkaline conditions by S_N_2 addition–elimination, ylide formation and C2-substitution
molecular dynamics (MD)	*ab initio* MD, all-atom MD	[166,172,176,188,190]	hydroxide ion solvation and diffusion mechanisms
mesoscale simulations	coarse-grained MD	[158,160]	microstructure, solvation and ion diffusivity
continuum modelling and simulation	three-dimensional agglomerate model, finite-volume modelling approach	[191,192]	inlet relative humidity, ionomer water uptake, platinum loading, carbon content and ionomer volume fraction

In general, computational modelling and simulation is a powerful technique for the investigation of biopolymer-based AEMFCs stability and transportation of OH^−^ ions. Moreover, it also enables us to the rational design of biopolymer-based AEMFCs structures with improved performance. Despite the low chemical stability of QA head groups, biopolymer-based AEM is still an interesting topic with great prospects owing to its advantages. Herein, further innovations in the QA head group structure, backbone architecture and biopolymer-based AEM design, guided by computational study is highly recommended to improve chemical stability and better transportation of OH^−^ ions in AEM to develop into large-scale application in fuel cells.

## 5. Conclusion

PEMs with Nafion being one of the most well-known examples, have been well-established and produced commercially for several decades. They are widely used in various applications, including fuel cells. By contrast, AEMs are still in the research and development phase and have not yet entered mass production. AEMs have the potential to be used in similar applications as PEMs but with some unique advantages, such as the ability to operate with non-precious metal catalysts and potentially lower costs. However, they face challenges related to stability, performance, and manufacturing processes that need to be addressed before they can be commercially viable.

The scientific community has not yet developed a globally recognized commercial standard for this particular use of fuel cells. However, various commercial membranes such as A201 membrane from Tokuyama Corporation (Japan), a Morgane ADP membrane from Solvay (Belgium), the FAA membrane from Fumatech (Germany), the Neosepta AHA from Tokuyama and the Tokuyama A006 are used for these purposes [14]. Comparison of prepared and commercial membranes in real AEMFCs is a practical and valuable method for assessing the potential of AEMs and their associated components. Testing AEMs in real AEMFCs allows for a comprehensive evaluation of the entire system, including the AEM itself, the electrocatalysts on the cathode and anode, and their interfaces. Only with this approach would be possible to compare the applicability of newly developed AEMs and commercial ones in alkaline AEMFCs, because the performance of an AEMFC depends not only on the individual components but also on how they interact within the system. To ensure meaningful comparisons between different AEMs, it is essential to use standardized testing conditions. This could include using a predefined fuel with a known composition, a consistent alkali content, and operating at a fixed temperature. For instance, power density is just one metric for assessing fuel cell performance. However, it is critical to recognize that variations in power density may not solely be owing to the membrane’s properties, and operating conditions also can play a significant role. Therefore, attributing low or high performance solely to the membrane can be misleading. To gain a better understanding of factors influencing fuel cell performance, a comprehensive testing strategy that considers various parameters and conditions is necessary.

CS-based polymers hold significant promise for a range of applications, including use in AEMFCs. AEMFCs are an emerging technology in the field of fuel cells, and they are being researched as an alternative to PEMFCs owing to several advantages, such as the use of non-precious metal catalysts and the ability to operate with a wide variety of fuels, including ammonia and biofuels. CS-based polymers have several characteristics that make them attractive for AEMFCs, and their future prospects in this field are promising. However, ongoing research and development efforts are necessary to address the remaining challenges and further optimize these materials for commercial applications in fuel cell technology. Challenges that need to be overcome include low ionic conductivity at higher pH and temperatures and insufficient long-term stability in alkaline conditions. To address these challenges, researchers have developed composite membranes using various techniques, including the use of nanostructured fillers, CNT, intercalated LDHs and two-dimensional laminates containing functional groups. As a result, hybrid approaches for creating electrolyte membranes have shown great potential in fuel cell technology. These approaches involve combining different materials or techniques to create a membrane that possesses the required characteristics, such as ionic conductivity, mechanical strength and chemical stability.

The review acknowledges that long-term alkaline stability and conductivity at high pH and temperatures are major challenges faced by researchers. To address these challenges, various approaches have been explored, including the use of modified nanoparticles, one-dimensional nanotubes, two-dimensional nanosheets with functional groups and grafting using macromolecules. These strategies aim to engineer composite membranes that can overcome the limitations of CS and exhibit improved performance under harsh operating conditions.

In summary, this review provides an overview of recent developments in modified CS as an AEM for fuel cells. It highlights the methods used for modification, the challenges faced in achieving long-term stability and high conductivity, and the potential of engineering composite membranes to overcome these challenges. The review also emphasizes the need for computational modelling and simulation that play a crucial role in understanding and optimizing the performance and durability of alkaline AEMFCs. By employing theoretical studies and modelling, researchers can gain fundamental insights into the electrochemical and transport processes occurring within these fuel cells, leading to performance improvements. However, to bridge the gap between theory and real-world performance, it is crucial to validate these models by comparing their predictions with experimental data obtained from operating fuel cells. By establishing a close relationship between membrane properties and fuel cell performance, researchers can develop advanced alkaline AEMFCs and optimize their fabrication and operation.

## Data Availability

This article has no additional data.
